# Prospective associations between sedentary time, physical activity, fitness and cardiometabolic risk factors in people with type 2 diabetes

**DOI:** 10.1007/s00125-015-3756-8

**Published:** 2015-10-30

**Authors:** Maxine J. E. Lamb, Kate Westgate, Søren Brage, Ulf Ekelund, Gráinne H. Long, Simon J. Griffin, Rebecca K. Simmons, Andrew J. M. Cooper, on behalf of the ADDITION-Plus study team

**Affiliations:** MRC Epidemiology Unit, University of Cambridge School of Clinical Medicine, Institute of Metabolic Science, Cambridge Biomedical Campus, Box 285, Cambridge, CB2 0QQ UK; Department of Sports Medicine, Norwegian School of Sport Sciences, Oslo, Norway; The Primary Care Unit, Institute of Public Health, University of Cambridge, Cambridge, UK

**Keywords:** Cardiometabolic, Cardiovascular, Diabetes, Fitness, Intervention, Physical activity, Prospective, Sedentary

## Abstract

**Aims/hypothesis:**

The aim of this study was to examine the prospective associations between objectively measured physical activity energy expenditure (PAEE), sedentary time, moderate-to-vigorous-intensity physical activity (MVPA), cardiorespiratory fitness (CRF) and cardiometabolic risk factors over 4 years in individuals with recently diagnosed diabetes.

**Methods:**

Among 308 adults (mean age 61.0 [SD 7.2] years; 34% female) with type 2 diabetes from the Anglo–Danish–Dutch Study of Intensive Treatment in People with Screen Detected Diabetes in Primary Care (ADDITION)-Plus study, we measured physical activity using individually calibrated combined heart rate and movement sensing. Multivariable linear regression models were constructed to examine the associations between baseline PAEE, sedentary time, MVPA, CRF and cardiometabolic risk factors and clustered cardiometabolic risk (CCMR) at follow-up, and change in these exposures and change in CCMR and its components over 4 years of follow-up.

**Results:**

Individuals who increased their PAEE between baseline and follow-up had a greater reduction in waist circumference (−2.84 cm, 95% CI −4.84, −0.85) and CCMR (−0.17, 95% CI −0.29, −0.04) compared with those who decreased their PAEE. Compared with individuals who decreased their sedentary time, those who increased their sedentary time had a greater increase in waist circumference (3.20 cm, 95% CI 0.84, 5.56). Increases in MVPA were associated with reductions in systolic blood pressure (−6.30 mmHg, 95% CI −11.58, −1.03), while increases in CRF were associated with reductions in CCMR (−0.23, 95% CI −0.40,−0.05) and waist circumference (−3.79 cm, 95% CI −6.62, −0.96). Baseline measures were generally not predictive of cardiometabolic risk at follow-up.

**Conclusions/interpretation:**

Encouraging people with recently diagnosed diabetes to increase their physical activity and decrease their sedentary time may have beneficial effects on their waist circumference, blood pressure and CCMR.

## Introduction

Individuals with type 2 diabetes are at increased risk of developing cardiovascular disease (CVD) and have poorer survival rates after diagnosis of CVD in comparison with those without diabetes [1–3]. Although physical inactivity is an important and well-established cardiometabolic risk factor [4, 5], individuals with diabetes are less likely to meet current physical activity guidelines [6, 7] compared with the general adult population.

In addition to the health benefits associated with increasing physical activity, recent research suggests that limiting the amount of time spent sedentary may also improve cardiometabolic health [8]. Several studies have demonstrated positive associations between objectively measured time spent sedentary and poor cardiometabolic outcomes, independent of moderate-to-vigorous-intensity physical activity (MVPA) in healthy adult populations [9–12]. Similarly, inverse associations between MVPA and cardiometabolic risk have been reported, independent of the time spent sedentary [13, 14]. Furthermore, while it is known that increases in physical activity are associated with increases in cardiorespiratory fitness (CRF), we have previously shown that the beneficial association between physical activity energy expenditure (PAEE; the energy expended above that required when resting) and metabolic risk factors is independent of CRF [15]. This is important because it means that increases in PAEE, even when below the level required to improve CRF, can still lead to important improvements in cardiometabolic health.

To our knowledge, no studies have examined the prospective association between CRF or objectively measured sedentary time and physical activity and metabolic risk among individuals with diabetes. Moreover, as studies have generally examined the associations between physical activity and cardiometabolic health cross-sectionally, the ability to infer the direction of these associations is limited.

Using data from the Anglo–Danish–Dutch Study of Intensive Treatment in People with Screen Detected Diabetes in Primary Care (ADDITION)-Plus study, we examined: (1) whether baseline objectively measured PAEE, sedentary time, MVPA and CRF were associated with cardiometabolic risk at 4 year follow-up; and (2) whether changes in these exposures were associated with changes in cardiometabolic risk over 4 years.

## Methods

### Study design and population

The rationale and design of the ADDITION-Plus study has previously been reported [16]. Briefly, ADDITION-Plus is an explanatory randomised controlled trial of a facilitator-led, theory-based behaviour change intervention tailored to individuals with recently diagnosed diabetes. Participants were recruited from 34 general practice clinics in East Anglia, UK. Eligible individuals were aged 40–69 years with screen-detected diabetes diagnosed through the ADDITION-Cambridge study [17] or clinically diagnosed in the previous 3 years. Women who were pregnant or lactating were excluded, as were individuals with a psychotic illness or a likely survival prognosis of less than 1 year.

Of the 1,109 eligible participants, 478 agreed to participate and were individually randomised to receive either intensive treatment alone (*n* = 239) or intensive treatment plus a facilitator-led individual behaviour change intervention targeting diet, physical activity, smoking and adherence to medication (*n* = 239). Measurements were carried out at baseline, 1 year and 5 years in outpatient clinical research facilities by trained staff following standard operating procedures. We used 1 year and 5 year follow-up data (hereafter referred to as baseline and follow-up) as objectively measured physical activity was measured only at these time points. As there were no between-group differences in health behaviours or CVD risk factors at 1 year [18], the trial arms were pooled and a cohort analysis was conducted. We excluded individuals who: (1) did not attend for follow-up (*n* = 80); (2) did not have complete data for physical activity and sedentary time at both baseline and follow-up (*n* = 49); and (3) did not have data for all cardiometabolic outcomes and covariates (*n* = 41). The final sample size for our study was 308 participants. All participants gave written informed consent and ethical approval was obtained from the Eastern Multi-Centre Research Ethics Committee (reference number 02/5/54).

### Measurement of physical activity, sedentary time and CRF

Free-living physical activity was measured using a combined heart rate and movement sensor (Actiheart, CamNtech, Cambridge, UK) worn continuously for ≥4 days, as described elsewhere [19]. A graded treadmill walk test was used to individually calibrate heart rate [20] and to estimate CRF in individuals who had at least a 10 min test duration by extrapolation of the heart rate/oxygen consumption relationship to the age-predicted maximum heart rate [21]. A group calibration equation adjusted for age, sex, β-blockers and sleeping heart rate was developed and used for the translation of heart rate into physical activity intensity in participants who did not complete an individual calibration test. Heart rate data collected during the free-living period were pre-processed [22] and the average activity intensity (J min^−1^ kg^−1^) was estimated using a branched equation framework [23]. The resulting intensity time-series data were summarised into PAEE (kJ kg^−1^ day^−1^), time spent sedentary (h/day), excluding self-reported sleep, and time spent in MVPA (min/day). Periods of non-wear were inferred from the combination of non-physiological heart rate (large Bayesian uncertainty [22]) and periods of inactivity (accelerometry counts of zero) lasting more than 90 min. Summary estimates were generated while minimising diurnal information bias caused by non-wear periods. Time spent sedentary was defined as a metabolic equivalent of task (MET) value of <1.5 [24] and MVPA as ≥3.0 METs, primarily using the Oxford estimate of resting metabolic rate to define 1.0 MET [25], and secondarily using a fixed value of 20.35 J ml O_2_ × 3.5 ml O_2_ min^−1^ kg^−1^. MVPA classified by a higher threshold of 3.5 METs was also investigated. All individuals included in this analysis had at least 3 days of valid wear time and 8 h of wear time per day.

### Measurement of cardiometabolic risk factors and covariates

Height and weight were measured with individuals wearing light clothing but no shoes, using a fixed rigid stadiometer and scale (SECA, Birmingham, UK). Waist circumference was measured as the mean of two measurements taken with a tape measure at the midpoint between the lowest point of the rib cage and the anterior superior iliac crest while standing. Blood pressure was measured as the mean of three measurements performed after 10 min of rest while participants were seated with a cuff placed on their dominant arm at the level of the heart, using an automated sphygmomanometer (Omron M4, Milton Keynes, UK). HbA_1c_ was measured in venous samples using an ion-exchange HPLC (Tosoh Bioscience, Redditch, UK). Serum HDL-cholesterol and triacylglycerol were measured using enzymatic techniques (Dade Behring Dimension Analyzer; Dade Behring, Newark, NJ, USA).

Standardised questionnaires were used to collect information on sociodemographic characteristics and lifestyle behaviours, including sleep times, smoking status, alcohol consumption and medication use. Alcohol consumption was reported as units per week. Smoking status was categorised as current, former or never smoker. Occupational socioeconomic class was categorised as managerial/professional, intermediate or routine/manual based on current or previous occupation. A validated food frequency questionnaire was used to estimate total daily energy intake [26].

### Calculation of the clustered cardiometabolic risk score

A clustered cardiometabolic risk (CCMR) score was constructed by summing *z* scores (units of SD from the population mean) of baseline values for waist circumference, systolic blood pressure (SBP), HbA_1c_, the inverse of HDL-cholesterol concentration and, due to its non-normal distribution, the natural log of triacylglycerol concentration, using sex-specific baseline means and SDs (CCMR = [value − mean]/SD), from which *z* scores of the follow-up variables were also computed. The use of a common mean and SD for standardised variables at two time points ensures that changes in the score can vary from zero. We divided both by 5, separately, to account for the number of variables included. Change in the CCMR was calculated by subtracting the follow-up CCMR from the baseline CCMR. To examine whether waist circumference was a mediator of any associations, a second score was made by excluding waist circumference from the CCMR (denoted CCMR_−WC_), thereby allowing waist circumference to be used as a covariate.

### Statistical analysis

Descriptive characteristics at baseline and follow-up were summarised separately for men and women. Paired *t* tests, *χ*^2^ tests and Wilcoxon signed rank tests were used to examine differences between individuals’ characteristics at baseline and follow-up. In addition, *t* tests or *χ*^2^ tests were used to investigate differences between individuals with and without missing data at both time points. Spearman correlation coefficients were calculated to examine the correlations between the baseline and follow-up values of the physical activity exposures.

We used multivariable linear regression analyses to model the associations between baseline PAEE, sedentary time, MVPA and CRF, and 4 year values of waist circumference, SBP, the natural log of triacylglycerol concentration, HDL-cholesterol level, HbA_1c_ and CCMR scores. Models were adjusted for age and sex (model 1), intervention group, occupational socioeconomic class and baseline smoking status, sleep duration, total energy intake, alcohol intake, waist circumference (except when waist circumference or CCMR was included as an outcome), use of antihypertensive, glucose-lowering or lipid-lowering drugs where appropriate and baseline levels of outcome variables (model 2). To investigate the independent associations of sedentary time and MVPA with each outcome, we additionally adjusted MVPA for sedentary time and vice versa (model 3). Next, we used multivariable linear regression analyses to examine the associations between changes from baseline to follow-up in PAEE, sedentary time, MVPA and CRF, with changes in CCMR and its components over the same period. Models were adjusted for the baseline and follow-up levels of the covariates listed above as well as the baseline levels of the exposure and outcome variables. All regression models were analysed with PAEE, sedentary time, MVPA and CRF as tertiles due to violation of the assumption of linearity between baseline physical activity/fitness variables and cardiometabolic risk variables at follow-up; however, our data met all other assumptions of linear regression.

We investigated interaction by sex by entering cross-product terms (i.e. MVPA × sex) with main effects in the most adjusted multivariable models. After confirming no effect modification by sex (all *p* values ≥0.05), except for the associations between sedentary time and waist circumference and between change in PAEE and change in waist circumference, we chose to conduct pooled analyses adjusted for sex.

All statistical analyses were performed using Stata/SE version 13.1 (Stata-Corp, College Station, TX). Statistical significance was set at *p* < 0.05.

## Results

Table 1 shows the characteristics of the study participants (*n* = 308) at baseline and follow-up, stratified by sex. The mean age of participants was 61.0 (SD 7.2) years at baseline. Men had higher PAEE and MVPA at both baseline and follow-up, compared with women. Between baseline and follow-up, PAEE decreased among women whereas sedentary time increased. Among men, MVPA and PAEE decreased from baseline to follow-up whereas sedentary time increased. Baseline measures of PAEE were strongly positively correlated with MVPA (*ρ* = 0.92) and strongly negatively correlated with sedentary time (*ρ* = −0.80). MVPA at baseline was strongly negatively correlated with sedentary time at baseline (*ρ* = −0.68). Baseline PAEE, sedentary time, MVPA and CRF measures were moderately correlated with the same measures at follow-up (*ρ* = 0.61, 0.55, 0.58 and 0.57, respectively).

**Table 1:** Characteristics of ADDITION-Plus participants at baseline and follow-up

	Men		Women	
	Baseline	Follow-up	Baseline	Follow-up
*n*	202	202	106	106
Age (years)	60.9 ± 7.2	64.8 ± 7.3**	61.3 ± 7.1	65.1 ± 7.1**
Height (cm)	174.8 ± 6.9	174.2 ± 7.0**	161.4 ± 6.9	160.9 ± 6.8**
Weight (kg)	96.1 ± 16.5	96.3 ± 17.8	83.2 ± 16.0	83.0 ± 16.2
BMI (kg/m^2^)	31.4 ± 5.0	31.7 ± 5.4	31.9 ± 5.5	32.1 ± 5.6
Waist circumference (cm)	110.8 ± 12.6	110.6 ± 13.3	103.4 ± 12.7	102.2 ± 12.8
SBP (mmHg)	132.2 ± 17.0	132.6 ± 15.7	124.7 ± 17.0	129.0 ± 17.4*
HbA_1c_ (%)	6.66 ± 0.96	6.97 ± 0.95**	6.56 ± 0.85	6.88 ± 0.73**
HbA_1c_ (mmol/mol)	49.3 ± 10.5	52.7 ± 10.4**	48.3 ± 9.3	51.8 ± 8.0**
Triacylglycerol (mmol/l)	1.70 (1.20–2.30)	1.60 (1.20–2.30)	1.60 (1.10–2.20)	1.50 (1.10–2.10)
HDL-cholesterol (mmol/l)	1.11 ± 0.27	1.21 ± 0.30**	1.32 ± 0.30	1.42 ± 0.31**
CCMR	−0.004 ± 0.53	−0.01 ± 0.53	0.001 ± 0.55	0.04 ± 0.54
Medication				
Glucose-lowering	98 (48.5)	153 (75.7)**	52 (49.1)	68 (64.2)**
Antihypertensive	150 (74.3)	161 (79.7)**	76 (71.7)	79 (74.5)**
Lipid-lowering	154 (76.2)	173 (85.6)**	85 (80.2)	89 (84.0)**
PAEE (kJ kg^−1^ day^−1^)	34.5 (22.3–47.6)	29.8 (19.9–40.2)**	26.0 (17.5–34.5)	23.5 (14.4–32.2)**
Sedentary time (h/day)	9.98 (8.18–11.7)	10.46 (9.16–11.86)**	9.61 (8.13–11.21)	10.50 (8.88–12.08)**
MVPA (min/day)	71.1 (37.4–129.4)	63.5 (29.5–110.6)*	47.5 (25.1–88.1)	49.9 (18.6–93.7)
CRF (ml O_2_ kg^−1^ min^−1^)^a^	36.5 ± 8.0	35.7 ± 8.7	30.0 ± 6.5	32.3 ± 9.7
Sleep duration (h/day)	8.01 ± 1.00	8.19 ± 1.04**	8.73 ± 0.95	8.78 ± 0.84
Total energy intake (kJ/day)	7,430 ± 1,938	7,262 ± 2,603	7,004 ± 1,978	6,801 ± 2,085
Alcohol intake (units/week)	6 (1–14)	4 (0–14)	2 (0–5)	1 (0–5)
Smoking status				
Current	24 (11.9)	23 (11.4)**	17 (16.0)	17 (16.0)**
Former	116 (57.4)	114 (56.4)	43 (40.6)	41 (38.7)
Never	62 (30.7)	65 (32.2)	46 (43.4)	48 (45.3)
Occupational socioeconomic class^b^				
Managerial/professional	98 (48.5)	–	34 (32.1)	–
Intermediate	41 (20.3)	–	33 (31.1)	–
Routine/manual	63 (31.2)	–	39 (36.8)	–

Data are mean ± SD, median (interquartile range) or *n* (%)

^a^CRF data were available for 92 men and 33 women

^b^Occupational socioeconomic class at study baseline

*p < 0.05, **p < 0.01 baseline vs follow-up, generated by paired *t* test for normally distributed data, Wilcoxon signed rank test for non-normally distributed data and *χ* ^2^ test for proportions

Compared with participants with complete data at baseline, women with missing data had a higher BMI and SBP and a larger waist circumference; men had only a larger waist circumference (all *p* ≤ 0.05). At follow-up, women with missing data had a higher a BMI and SBP, and men had a higher HbA_1c_, compared with participants with complete data (all *p* ≤ 0.01). CRF data were present for 125 participants. These individuals had lower BMI and waist circumference at baseline and follow-up (*p* < 0.01) than the rest of the study population. Those with CRF data also had a higher PAEE and MVPA at both baseline and follow-up and spent less time sedentary at both time points (*p* < 0.001).

In adjusted analyses (models 2 and 3), the baseline time spent sedentary and MVPA were not predictive of CCMR or individual cardiometabolic risk factors at follow-up (Table 2). Unexpectedly, those in the highest tertile for CRF had a 9.03 mmHg (95% CI 3.17, 14.89 mmHg) higher SBP than those in the lowest tertile. When compared with individuals in the lowest tertile for PAEE at baseline, those in the highest tertile had a 0.42% (95% CI 0.12%, 0.73%) higher HbA_1c_ at follow-up. No other statistically significant associations were found.

**Table 2:** Adjusted associations between baseline physical activity, sedentary time and CRF and cardiometabolic risk factors and CCMR at 4 year follow-up in the ADDITION-Plus cohort

	Model 1			Model 2			Model 3		
	T1	T2	T3	T1	T2	T3	T1	T2	T3
PAEE (kJ kg^−1^ day^−1^)									
CCMR	Ref	−0.10 (−0.28, 0.08)	−0.18 (−0.37, 0.00)	Ref	0.01 (−0.13, 0.15)	−0.03 (−0.18, 0.12)			
CCMR_−WC_	Ref	−0.03 (−0.22, 0.16)	−0.04 (−0.23, 0.16)	Ref	0.03 (−0.12, 0.19)	0.02 (−0.15, 0.19)			
WC (cm)	Ref	−4.92 (−9.31, −0.53)*	−9.60 (−14.10, −5.11)*	Ref	−0.31 (−2.54, 1.92)	0.85 (−1.54, 3.23)			
TG (mmol/l)	Ref	−0.04 (−0.23, 0.15)	−0.01 (−0.20, 0.18)	Ref	0.10 (−0.04, 0.24)	0.03 (−0.12, 0.18)			
SBP (mmHg)	Ref	−2.64 (−7.97, 2.69)	−6.46 (−11.91, −1.00)*	Ref	−3.74 (−8.70, 1.22)	−6.79 (−12.14, −1.44)*			
HbA_1c_ (%)	Ref	0.23 (−0.08, 0.54)	0.35 (0.03, 0.67)*	Ref	0.21 (−0.08, 0.49)	0.42 (0.12, 0.73)*			
HbA_1c_ (mmol/mol)	Ref	2.57 (−0.82, 5.95)	3.84 (0.37, 7.30)*	Ref	2.27 (−0.84, 5.39)	4.63 (1.27, 7.98)*			
HDL (mmol/l)	Ref	0.01 (−0.10, 0.11)	0.02 (−0.09, 0.12)	Ref	−0.05 (−0.12, 0.02)	−0.04 (−0.12, 0.03)			
Sedentary time (h/day)									
CCMR	Ref	−0.09 (−0.23, 0.06)	0.09 (−0.06, 0.23)	Ref	−0.04 (−0.15, 0.07)	−0.03 (−0.14, 0.09)	Ref	−0.03 (−0.15, 0.09)	−0.01 (−0.15, 0.13)
CCMR_−WC_	Ref	−0.11 (−0.27, 0.04)	0.03 (−0.13, 0.18)	Ref	−0.05 (−0.17, 0.08)	−0.02 (−0.15, 0.11)	Ref	−0.02 (−0.16, 0.11)	0.02 (−0.14, 0.18)
WC (cm)	Ref	0.10 (−3.48, 3.68)	4.20 (0.61, 7.79)*	Ref	−0.48 (−2.20, 1.24)	−1.76 (−3.59, 0.08)	Ref	−0.69 (−2.57, 1.19)	−2.13 (−4.41, 0.14)
TG (mmol/l)	Ref	−0.03 (−0.17, 0.11)	−0.02 (−0.16, 0.13)	Ref	0.05 (−0.06, 0.15)	−0.03 (−0.14, 0.08)	Ref	0.04 (−0.07, 0.16)	−0.04 (−0.18, 0.10)
SBP (mmHg)	Ref	−1.23 (−5.66, 3.20)	2.95 (−1.50, 7.39)	Ref	−1.56 (−5.56, 2.43)	2.72 (−1.55, 6.99)	Ref	−2.17 (−6.52, 2.19)	1.66 (−3.59, 6.91)
HbA_1c_ (%)	Ref	−0.10 (−0.34, 0.13)	−0.16 (−0.39, 0.08)	Ref	−0.13 (−0.33, 0.07)	−0.20 (−0.41, 0.02)	Ref	−0.03 (−0.25, 0.18)	−0.01 (−0.28, 0.25)
HbA_1c_ (mmol/mol)	Ref	−1.13 (−3.69, 1.42)	−1.70 (−4.26, 0.86)	Ref	−1.47 (−3.66, 0.72)	−2.13 (−4.47, 0.20)	Ref	−0.35 (−2.72, 2.02)	−0.15 (−3.02, 2.72)
HDL (mmol/l)	Ref	0.07 (−0.01, 0.15)	−0.03 (−0.11, 0.06)	Ref	0.01 (−0.04, 0.07)	0.02 (−0.04, 0.07)	Ref	0.01 (−0.05, 0.07)	0.02 (−0.05, 0.09)
MVPA (min/day)									
CCMR	Ref	−0.03 (−0.18, 0.12)	−0.07 (−0.22, 0.08)	Ref	0.06 (−0.05, 0.17)	0.08 (−0.03, 0.20)	Ref	0.09 (−0.03, 0.21)	0.14 (−0.02, 0.29)
CCMR_-WC_	Ref	−0.01 (−0.16, 0.15)	0.01 (−0.15, 0.16)	Ref	0.07 (−0.06, 0.19)	0.10 (−0.03, 0.23)	Ref	0.09 (−0.04, 0.23)	0.15 (−0.03, 0.33)
WC (cm)	Ref	−1.51 (−5.11, 2.09)	−4.89 (−8.57, −1.22)*	Ref	0.17 (−1.56, 1.90)	0.98 (−0.81, 2.78)	Ref	0.10 (−1.86, 2.06)	0.85 (−1.64, 3.35)
TG (mmol/l)	Ref	−0.01 (−0.15, 0.14)	0.02 (−0.12, 0.17)	Ref	0.05 (−0.05, 0.16)	0.05 (−0.06, 0.16)	Ref	0.04 (−0.08, 0.16)	0.02 (−0.13, 0.17)
SBP (mmHg)	Ref	1.44 (−3.01, 5.89)	−3.54 (−8.08, 1.00)	Ref	0.76 (−3.29, 4.81)	−2.36 (−6.59, 1.87)	Ref	2.70 (−1.82, 7.21)	1.39 (−4.38, 7.17)
HbA_1c_ (%)	Ref	0.06 (−0.17, 0.29)	0.24 (0.00, 0.48)	Ref	0.11 (−0.09, 0.31)	0.30 (0.09, 0.51)*	Ref	0.08 (−0.15, 0.30)	0.23 (−0.06, 0.52)
HbA_1c_ (mmol/mol)	Ref	0.66 (−1.90, 3.22)	2.61 (0.00, 5.22)	Ref	1.25 (−0.94, 3.43)	3.27 (1.00, 5.53)	Ref	0.86 (−1.61, 3.32)	2.53 (−0.62, 5.67)
HDL (mmol/l)	Ref	0.05 (−0.03, 0.14)	0.02 (−0.06, 0.11)	Ref	0.00 (−0.05, 0.06)	−0.03 (−0.09, 0.02)	Ref	0.03 (−0.06, 0.12)	−0.04 (−0.12, 0.04)
CRF (ml O_2_ kg^−1^ min^−1^)^a^									
CCMR	Ref	0.14 (−0.08, 0.35)	−0.05 (−0.27, 0.17)	Ref	0.07 (−0.09, 0.23)	0.05 (−0.12, 0.21)			
CCMR_−WC_	Ref	0.18 (−0.05, 0.41)	0.05 (−0.18, 0.28)	Ref	0.10 (−0.08, 0.28)	0.11 (−0.08, 0.30)			
WC (cm)	Ref	−0.34 (−5.62, 4.93)	−5.61 (−11.02, −0.19)*	Ref	0.10 (−2.47, 2.66)	−0.54 (−3.26, 2.19)			
TG (mmol/l)	Ref	−0.01 (−0.21, 0.20)	−0.06 (−0.27, 0.15)	Ref	0.06 (−0.09, 0.22)	0.05 (−0.12, 0.22)			
SBP (mmHg)	Ref	11.05 (4.91, 17.19)*	6.29 (−0.02, 12.60)	Ref	10.61 (5.01, 16.21)*	9.03 (3.17, 14.89)*			
HbA_1c_ (%)	Ref	−0.03 (−0.43, 0.38)	−0.03 (−0.45, 0.39)	Ref	−0.19 (−0.52, 0.13)	−0.06 (−0.40, 0.28)			
HbA_1c_ (mmol/mol)	Ref	−0.31 (−4.75, 4.14)	−0.34 (−4.91, 4.23)	Ref	−2.12 (−5.64, 1.40)	−0.63 (−4.36, 3.09)			
HDL (mmol/l)	Ref	−0.05 (−0.18, 0.08)	0.00 (−0.13, 0.14)	Ref	−0.01 (−0.10, 0.08)	0.03 (−0.07, 0.12)			

Data are unstandardised regression coefficients and 95% CI

Model 1: adjusted for age and sex

Model 2: model 1 + intervention group, occupational socioeconomic class, smoking status, sleep duration, total energy intake, alcohol intake and WC (except when WC or CCMR are outcomes). SBP additionally adjusted for antihypertensive drugs at baseline. HbA_1c_ adjusted for glucose-lowering drugs at baseline. HDL-cholesterol adjusted for lipid-lowering drugs at baseline. CCMR adjusted for all three classes of drugs at baseline

Model 3: model 2 + MVPA (when examining sedentary time) or sedentary time (when examining MVPA)

CCMR was constructed by summing sex-specific values for WC, SBP, HbA_1c_, the inverse of HDL and the natural log of TG, using sex-specific means and SDs ((CCMR = [value − mean]/SD)/5)

^a^ *n* = 125

*p < 0.05

T1–T3, tertiles 1–3; Ref, Reference; WC, waist circumference; TG, triacylglycerol

Individuals in the lowest, middle or highest sex-specific tertiles of change for PAEE, sedentary time, MVPA and CRF decreased, maintained or increased their levels, respectively, over the 4 years of follow-up (Table 3). The lowest, middle and highest tertiles of change are therefore referred to as ‘decreasers’, ‘maintainers’ and ‘increasers’, respectively. Participants who increased their PAEE and CRF between baseline and follow-up had greater reductions in CCMR and waist circumference than decreasers (Table 3). Those who increased their sedentary time had a 3.20 cm (95% CI 0.84, 5.56 cm) greater increase in waist circumference than decreasers. Individuals who increased their MVPA had a 6.30 mmHg (95% CI 1.03, 11.58 mmHg) greater reduction in SBP compared with decreasers. When we removed waist circumference from the CCMR score to examine mediation by waist circumference, the association between change in PAEE and change in CCMR_−WC_ was not significant (Table 3). No other statistically significant associations were found (Table 3).

**Table 3:** Adjusted associations between change in physical activity, sedentary time and CRF and change in cardiometabolic risk factors and CCMR over 4 years in the ADDITION-Plus cohort

	Model 1			Model 2			Model 3		
	Decreasers	Maintainers	Increasers	Decreasers	Maintainers	Increasers	Decreasers	Maintainers	Increasers
PAEE (kJ kg^−1^ day^−1^)									
CCMR	Ref	−0.05 (−0.16, 0.07)	−0.18 (−0.30, −0.07)*	Ref	−0.06 (−0.18, 0.06)	−0.17 (−0.29, −0.04)*			
CCMR_−WC_	Ref	−0.02 (−0.15, 0.11)	−0.17 (−0.31, −0.04)*	Ref	0.00 (−0.13, 0.13)	−0.10 (−0.24, 0.04)			
WC (cm)	Ref	−1.76 (−3.47, −0.06)*	−2.77 (−4.48, −1.06)*	Ref	−2.05 (−3.93, −0.17)*	−2.84 (−4.84, −0.85)*			
TG (mmol/l)	Ref	0.13 (−0.13, 0.39)	−0.07 (−0.33, 0.19)	Ref	0.12 (−0.14, 0.39)	−0.06 (−0.34, 0.22)			
SBP (mmHg)	Ref	−2.67 (−7.21, 1.87)	−3.36 (−7.91, 1.19)	Ref	−1.44 (−5.78, 2.90)	−2.22 (−6.84, 2.39)			
HbA_1c_ (%)	Ref	−0.07 (−0.31, 0.17)	−0.20 (−0.44, 0.04)	Ref	−0.01 (−0.23, 0.21)	−0.04 (−0.27, 0.19)			
HbA_1c_ (mmol/mol)	Ref	0.77 (−1.85, 3.39)	2.23 (−0.39, 4.86)	Ref	0.12 (−2.30, 2.55)	0.43 (−2.12, 2.98)			
HDL (mmol/l)	Ref	−0.01 (−0.07, 0.04)	0.04 (−0.01, 0.10)	Ref	−0.02 (−0.08, 0.04)	0.01 (−0.05, 0.08)			
Sedentary time (h/day)									
CCMR	Ref	0.12 (0.01, 0.24)*	0.08 (−0.03, 0.20)	Ref	0.12 (0.00, 0.23)	0.09 (−0.04, 0.22)	Ref	0.09 (−0.03, 0.21)	0.04 (−0.11, 0.18)
CCMR_−WC_	Ref	0.12 (−0.02, 0.25)	0.05 (−0.08, 0.18)	Ref	0.06 (−0.06, 0.19)	−0.00 (−0.15, 0.14)	Ref	0.03 (−0.10, 0.16)	−0.06 (−0.23, 0.10)
WC (cm)	Ref	1.90 (0.20, 3.61)*	2.74 (1.03, 4.45)*	Ref	2.31 (0.53, 4.09)*	3.32 (1.28, 5.35)*	Ref	2.25 (0.38, 4.12)*	3.20 (0.84, 5.56)*
TG (mmol/l)	Ref	0.02 (−0.23, 0.28)	0.02 (−0.24, 0.28)	Ref	−0.02 (−0.27, 0.24)	−0.06 (−0.36, 0.23)	Ref	−0.02 (−0.29, 0.25)	−0.07 (−0.40, 0.27)
SBP (mmHg)	Ref	1.96 (−2.59, 6.52)	−0.28 (−4.84, 4.28)	Ref	−0.36 (−4.52, 3.79)	−1.17 (−5.93, 3.59)	Ref	−1.71 (−6.04, 2.62)	−3.98 (−9.43, 1.46)
HbA_1c_ (%)	Ref	0.07 (−0.17, 0.31)	0.08 (−0.16, 0.33)	Ref	0.09 (−0.12, 0.30)	0.09 (−0.15, 0.34)	Ref	0.07 (−0.16, 0.29)	0.04 (−0.24, 0.32)
HbA_1c_ (mmol/mol)	Ref	−0.73 (−3.37, 1.90)	−0.92 (−3.56, 1.72)	Ref	−0.99 (−3.31, 1.33)	−1.02 (−3.70, 1.65)	Ref	−0.72 (−3.15, 1.71)	−0.46 (−3.53, 2.61)
HDL (mmol/l)	Ref	−0.04 (−0.10, 0.02)	−0.01 (−0.07, 0.04)	Ref	−0.03 (−0.08, 0.03)	0.01 (−0.06, 0.07)	Ref	−0.02 (−0.08, 0.04)	0.02 (−0.06, 0.09)
MVPA (min/day)									
CCMR	Ref	−0.03 (−0.14, 0.09)	−0.15 (−0.26, −0.03)*	Ref	0.01 (−0.12, 0.13)	−0.12 (−0.24, 0.00)	Ref	−0.00 (−0.13, 0.12)	−0.14 (−0.29, 0.00)
CCMR_−WC_	Ref	−0.01 (−0.15, 0.12)	−0.14 (−0.28, −0.01)*	Ref	0.02 (−0.11, 0.16)	−0.08 (−0.22, 0.06)	Ref	−0.00 (−0.14, 0.14)	−0.13 (−0.29, 0.03)
WC (cm)	Ref	−0.89 (−2.61, 0.83)	−2.10 (−3.82, −0.37)*	Ref	−0.58 (−2.56, 1.40)	−1.82 (−3.78, 0.14)	Ref	−0.23 (−2.29, 1.83)	−1.02 (−3.36, 1.33)
TG (mmol/l)	Ref	0.05 (−0.21, 0.31)	−0.04 (−0.30, 0.22)	Ref	0.09 (−0.18, 0.37)	−0.04 (−0.31, 0.24)	Ref	0.11 (−0.18, 0.39)	−0.00 (−0.33, 0.32)
SBP (mmHg)	Ref	−1.03 (−5.57, 3.51)	−3.99 (−8.54, 0.56)	Ref	−1.48 (−5.97, 3.01)	−3.23 (−7.71, 1.26)	Ref	−2.89 (−7.53, 1.75)	−6.30 (−11.58, −1.03)*
HbA_1c_ (%)	Ref	−0.12 (−0.36, 0.12)	−0.14 (−0.39, 0.10)	Ref	−0.01 (−0.23, 0.22)	−0.03 (−0.26, 0.20)	Ref	−0.01 (−0.25, 0.23)	−0.04 (−0.31, 0.24)
HbA_1c_ (mmol/mol)	Ref	1.29 (−1.34, 3.92)	1.58 (−1.06, 4.22)	Ref	0.06 (−2.44, 2.56)	0.35 (−2.14, 2.84)	Ref	0.08 (−2.53, 2.70)	0.40 (−2.57, 3.36)
HDL (mmol/l)	Ref	−0.03 (−0.08, 0.03)	0.03 (−0.03, 0.09)	Ref	−0.03 (−0.09, 0.03)	0.01 (−0.05, 0.07)	Ref	−0.03 (−0.10, 0.03)	0.00 (−0.07, 0.08)
CRF (ml O_2_ kg^−1^ min^−1^)^a^									
CCMR	Ref	−0.07 (−0.23, 0.08)	−0.21 (−0.36, −0.05)*	Ref	−0.07 (−0.24, 0.10)	−0.23 (−0.40, −0.05)*			
CCMR_−WC_	Ref	−0.04 (−0.23, 0.14)	−0.21 (−0.39, −0.02)*	Ref	0.03 (−0.17, 0.22)	−0.12 (−0.33, 0.08)			
WC (cm)	Ref	−2.45 (−4.91, −0.00)*	−2.64 (−5.12, −0.17)*	Ref	−2.78 (−5.48, −0.08)*	−3.79 (−6.62, −0.96)*			
TG (mmol/l)	Ref	−0.09 (−0.50, 0.32)	−0.39 (−0.80, 0.02)	Ref	0.14 (−0.27, 0.55)	−0.11 (−0.53, 0.32)			
SBP (mmHg)	Ref	−5.08 (−11.66, 1.51)	−8.56 (−15.19, −1.92)*	Ref	−1.07 (−7.04, 4.90)	−5.34 (−11.57, 0.90)			
HbA_1c_ (%)	Ref	0.08 (−0.27, 0.42)	−0.09 (−0.44, 0.26)	Ref	0.08 (−0.27, 0.42)	0.10 (−0.26, 0.45)			
HbA_1c_ (mmol/mol)	Ref	−0.86 (−4.63, 2.90)	1.01 (−2.79, 4.81)	Ref	−0.84 (−4.61, 2.93)	−1.05 (−4.91, 2.81)			
HDL (mmol/l)	Ref	−0.02 (−0.11, 0.06)	0.02 (−0.07, 0.10)	Ref	0.00 (−0.10, 0.09)	0.04 (−0.06, 0.13)			

Data are unstandardised regression coefficients and 95% CI

Model 1: adjusted for age at baseline and sex

Model 2: model 1 + intervention group, occupational socioeconomic class at baseline, baseline values of the respective exposure and outcome and change in age, smoking status, sleep duration, total energy intake, alcohol intake, WC and medication use (antihypertensive, lipid-lowering and glucose-lowering)

Model 3: model 2 + change in MVPA (when examining sedentary time) or change in sedentary time (when examining MVPA)

Change by tertile (mean ± SD):

PAEE (kJ kg^−1^ day^−1^): decreasers, −20.19 ± 10.30; maintainers, −4.1 ± 3.08; increasers, 10.04 ± 7.69

Sedentary time (h/day): decreasers, −1.76 ± 1.24; maintainers, 0.66 ± 0.57; increasers, 2.93 ± 1.28

MVPA (min/day): decreasers, −73.90 ± 52.90; maintainers, −5.23 ± 9.85; increasers, 54.44 ± 41.75

CRF (ml O_2_ kg^−1^ min^−1^): decreasers, −8.05 ± 6.19; maintainers, −0.13 ± 2.20; increasers, 8.49 ± 5.64

CCMR was constructed by summing sex-specific values for WC, SBP, HbA_1c_, the inverse of HDL and the natural log of TG, using sex-specific means and SDs ((CCMR = [value − mean]/SD)/5)

^a^ *n* = 125

*p < 0.05

Ref, reference; WC, waist circumference; TG, triacylglycerol

**Fig. 1:**
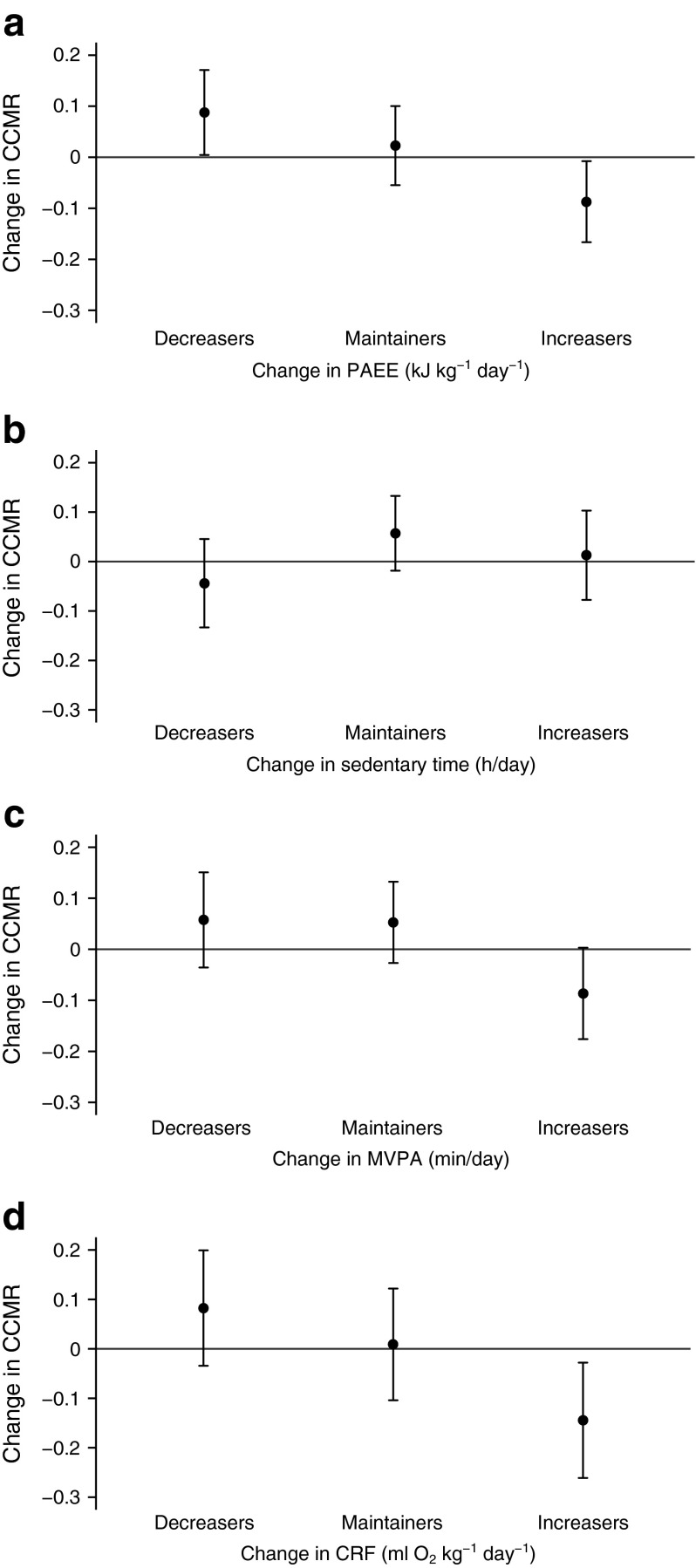
Adjusted mean change in physical activity, sedentary time and CRF and change in CCMR, by tertiles of change in physical activity. Data are means and 95% CIs adjusted for age, sex, intervention group, occupational socioeconomic class, clustered metabolic risk score and physical activity variable at baseline and change in age, alcohol consumption, energy intake, smoking status, sleep duration and medication use. (**b**) and (**c**) are additionally adjusted for change in MVPA and change in sedentary time, respectively. *p*_trend_: (**a**) 0.009; (**b**) 0.47; (**c**) 0.05; (**d**) 0.01

Figure 1 shows the adjusted mean change in CCMR by tertiles of change in PAEE, sedentary time, MVPA and CRF. Those who increased their PAEE, MVPA and/or CRF had the smallest increase in CCMR over the 4 year follow-up (PAEE, *p* value for trend [*p*_trend_] = 0.01; MVPA, *p*_trend_ = 0.05; CRF, *p*_trend_ = 0.01). Conversely, those who increased their sedentary time had the greatest increase in CCMR, although this trend was not statistically significant (*p*_trend_ = 0.47).

When the associations between baseline sedentary time and waist circumference at follow-up were stratified by sex, no associations were observed in men (highest vs lowest tertiles: −2.33 cm, 95% CI −5.21, 0.54) or women (highest vs lowest tertiles: −1.61 cm, 95% CI −5.59, 2.37). When associations between change in PAEE and change in waist circumference were examined by sex, the association was not significant in men (increasers vs decreasers: −2.06 cm, 95% CI −4.63, 0.51 cm) but was stronger in women (increasers vs decreasers: −4.42 cm, 95% CI −7.69, −1.14 cm; *p* for interaction = 0.10). Use of a fixed intensity threshold to define MVPA did not change the results except for a significant inverse association between change in PAEE and change in waist circumference (−2.25 cm, 95% CI −4.25, −0.24 cm). Increasing the threshold of time spent in MVPA to 3.5 METs caused the association between change in MVPA and change in CCMR to become statistically significant, although the point estimate did not change (increasers vs decreasers: −0.14, 95% CI −0.25, −0.02). Associations with the other risk factors remained very similar (data not shown).

## Discussion

In this prospective study of men and women with recently diagnosed type 2 diabetes, we show that individuals who increased their level of MVPA experienced a large and clinically meaningful reduction in SBP, and those who increased their PAEE or CRF had clinically significant improvements in waist circumference and CCMR. We additionally show that individuals who increased the duration of time spent sedentary had the greatest increase in waist circumference, independent of time spent in MVPA and baseline waist circumference. Baseline values for PAEE, MVPA and CRF were generally not predictive of cardiometabolic risk factors at follow-up. Our findings suggest that increasing the amount of time spent being physically active and decreasing the time spent sedentary may be an important strategy for self-management of diabetes early in the course of the disease.

Important strengths of our study include the prospective, population-based study design and use of objective measures of free-living physical activity at two time points, which enabled us to examine the magnitude of the longer term associations between changes in levels of physical activity and time spent sedentary, and changes in a number of cardiometabolic risk factors. Additionally, as only a small proportion of individuals were lost to follow-up and physical activity and cardiometabolic risk factor data were available for most individuals at both time points, our findings are likely to be generalisable to the original cohort of ADDITION-Plus participants.

Several limitations of our study also warrant discussion. As our study included a relatively homogenous population of older white adults, our results may not be generalisable to younger and more ethnically diverse populations. Although individuals with complete data did not differ on important cardiovascular risk factors, they did have a lower BMI and waist circumference compared with those with incomplete data. We conducted multiple hypothesis tests so we cannot exclude chance as an explanation for some of our findings, especially regarding the observed associations between baseline CRF and SBP, and PAEE and HbA_1c_, neither of which was replicated in the analyses investigating change. Finally, although we adjusted our analyses for a comprehensive range of potential confounders, we cannot exclude the possibility of residual confounding or confounding by unmeasured or unknown factors.

We used objective measures of physical activity and sedentary time, which allowed us to more accurately assess physical activity compared with self-report measures. By using individually calibrated combined heart rate and movement monitoring, we were able to include non-ambulatory activities such as cycling, which would be difficult to detect if we had used only a waist-mounted accelerometer. However, piezo-electric accelerometers worn on the torso are not ideally suited to detect transitions between sitting and standing [27], which may have different physiological effects [28]. Our exposure measure is therefore unlikely to be very sensitive to differences in posture, and we also did not examine breaks in sedentary time, which have been shown to be associated with waist circumference [10, 29].

We adjusted for MVPA in the analyses of sedentary time and vice versa when examining intensity associations, so were able to investigate the independent contributions of these exposures. Although the mean MVPA in our study population may be considered high compared with that observed in other studies, this is likely to be due to how physical activity was measured in our study: we used an epoch frequency of 30 s, enabling relatively short periods of activity to be detected, and we used a relative definition of 3 METs, which averaged 168.2 and 149.8 J min^−1^ kg^−1^ for men and women, respectively. Furthermore, we were able to capture non-ambulatory physical activities that may not have been detected in studies using accelerometers alone. Our findings of a relatively high amount of accumulated MVPA is similar to what Hansen et al observed when using the same physical activity monitor as we used [30].

Previous cross-sectional studies have found inconsistent associations between objectively measured physical activity and sedentary time with cardiometabolic risk factors in the general adult population [9, 12, 14, 31] and in populations at high risk of diabetes [11, 32]. In a cross-sectional study of the ADDITION-Plus cohort at 1 year, we previously showed that CRF and PAEE were inversely associated with CCMR and waist circumference [33]. In a cross-sectional analysis of a different cohort of individuals with newly diagnosed diabetes, sedentary time was found to be positively associated with waist circumference; however, the authors found no associations between sedentary time at baseline or change in sedentary time over 6 months of follow-up and any of the measured cardiometabolic risk factors at 6 months follow-up [29].

Few prospective studies have examined associations between objectively measured physical activity, fitness or sedentary time and cardiometabolic risk [29, 15, 34–37]. Ekelund et al, in a general adult population (*n* = 258), found that an increase in PAEE over 5 years of follow-up was associated with reductions in fasting glucose, insulin, triacylglycerol and CCMR [15]. In a large international, multicentre study including 4,345 older men and women with impaired glucose tolerance, baseline ambulatory activity and change in ambulatory activity, assessed by pedometer, were both inversely associated with risk of cardiovascular events over 6 years of follow-up [37]. In a sample of 321 individuals with a family history of diabetes, an increase in total daily activity, measured by accelerometry, and aerobic fitness were associated with decreases in CCMR score after 1 year, although the change in PAEE measured using individually calibrated heart rate monitoring was not [34]. In the same population, but with 6 years of follow-up data, increases in MVPA were found to be associated with reductions in waist circumference and CCMR, while increases in time spent sedentary were associated with increases in waist circumference and CCMR [36]. However, these associations were attenuated following mutual adjustment for time spent sedentary and in MVPA. In addition, Lahjibi et al, in a general adult population, found no associations between sedentary time, measured by accelerometry, and cardiovascular risk factors over 3 years of follow-up [38].

The potential consequences of spending too much time sedentary has become an area of increased research focus. The physiological effects of prolonged sedentary time may be distinct from, and therefore independent of, those underlying physical activity [28]. As the majority of the woken day is spent sedentary, especially among individuals close to retirement age (the mean age of our population at follow-up was 65 years), there are clear windows of opportunity for increasing activity, the benefits of which can lead to clinically important improvements in cardiometabolic risk. Although the effects of the transition to retirement on physical activity levels remain unclear [39], small lifestyle changes such as replacing a short car journey with walking or cycling and taking the stairs instead of using an elevator present clear opportunities to counteract the potential decline in physical activity and increase in sedentary time due to retirement [40].

## Conclusion

In this population of older adults with diabetes, increases in PAEE and CRF over 4 years of follow-up were associated with reductions in CCMR, and increases in MVPA were associated with reductions in SBP, whereas increases in sedentary time were associated with increases in waist circumference. Further follow-up is necessary to establish whether changes in physical activity predict CVD events. Nevertheless, these data highlight the importance of encouraging patients to increase their physical activity and decrease their sedentary time.

## Abbreviations

ADDITION: Anglo–Danish–Dutch Study of Intensive Treatment in People with Screen Detected Diabetes in Primary Care
CCMR: Clustered cardiometabolic risk
CCMR_-WC_: Clustered cardiometabolic risk excluding waist circumference
CRF: Cardiorespiratory fitness
CVD: Cardiovascular disease
MET: Metabolic equivalent of task
MVPA: Moderate-to vigorous-intensity physical activity
PAEE: Physical activity energy expenditure
SBP: Systolic blood pressure
